# Net energy content of rice bran, corn germ meal, corn gluten feed, peanut meal, and sunflower meal in growing pigs

**DOI:** 10.5713/ajas.17.0829

**Published:** 2018-03-02

**Authors:** Yakui Li, Zhongchao Li, Hu Liu, Jean Noblet, Ling Liu, Defa Li, Fenglai Wang, Changhua Lai

**Affiliations:** 1 State Key Laboratory of Animal Nutrition, Ministry of Agriculture Feed Industry Centre, China Agricultural University, Beijing 100193, China; 2 The College of Animal Science and Technology, Hebei North University, Zhangjiakou, Hebei 075000, China; 3 INRA, UMR Pegase, 35590 Saint-Gilles, France

**Keywords:** By-product, Growing Pig, Heat Production, Indirect Calorimetry, Net Energy

## Abstract

**Objective:**

The objective of this experiment was to determine the net energy (NE) content of full-fat rice bran (FFRB), corn germ meal (CGM), corn gluten feed (CGF), solvent-extracted peanut meal (PNM), and dehulled sunflower meal (SFM) fed to growing pigs using indirect calorimetry or published prediction equations.

**Methods:**

Twelve growing barrows with an average initial body weight (BW) of 32.4±3.3 kg were allotted to a replicated 3×6 Youden square design with 3 successive periods and 6 diets. During each period, pigs were individually housed in metabolism crates for 16 d, which included 7 days for adaptation. On d 8, the pigs were transferred to the respiration chambers and fed one of the 6 diets at 2.0 MJ metabolizable energy (ME)/kg BW^0.6^/d. Total feces and urine were collected and daily heat production was measured from d 9 to d 13. On d 14 and d15, pigs were fed at their maintenance energy requirement level. On the last day pigs were fasted and fasting heat production was measured.

**Results:**

The NE of FFRB, CGM, CGF, PNM, and SFM measured by indirect calorimetry method was 12.33, 8.75, 7.51, 10.79, and 6.49 MJ/kg dry matter (DM), respectively. The NE/ME ratios ranged from 67.2% (SFM) to 78.5% (CGF). The NE values for the 5 ingredients calculated according to the prediction equations were 12.22, 8.55, 6.79, 10.51, and 6.17 MJ/kg DM, respectively.

**Conclusion:**

The NE values were the highest for FFRB and PNM and the lowest in the corn co-products and SFM. The average NE of the 5 ingredients measured by indirect calorimetry method in the current study was greater than values predicted from NE prediction equations (0.32 MJ/kg DM).

## INTRODUCTION

In the past few years, the prices of corn and soybean meal, the two most commonly used energy and protein ingredients for swine, have risen dramatically. Less-expensive, alternative feed ingredients rich in dietary fiber or in protein have been increasingly included in swine diets in order to decrease feed costs. One example is full-fat rice bran (FFRB), a by-product of the rice grain milling process, which is a source of energy due to its high lipid content. Corn germ meal (CGM) and corn gluten feed (CGF) are two important corn co-products of the wet milling industry for starch or ethanol production. Peanut meal (PNM, solvent extracted) and sunflower meal (SFM, dehulled) are alternative oilseed meals for swine.

Several research projects have been conducted to evaluate the nutrient content and the digestibility of energy of FFRB [1], CGM and CGF [2,3], PNM [4], and SFM [5,6]. However, there is a lack of information about the measured net energy (NE) content of these ingredients and the NE values of these ingredients that are available are based on prediction equations using digestible energy (DE), metabolizable energy (ME) or digestible nutrients contents [7]. Prediction equations are used to estimate the NE content in swine diets and their use is supported by the results of some recent studies [8,9], however, others have reported differences between values obtained with prediction equations and those determined using the indirect calorimetry (IC) method [10,11]. Therefore, there is a need to further evaluate the NE values of these ingredients using prediction equations and empirical measurements so as to guide their use in formulating swine diets. Thus, the objectives of our study were to determine the NE content of FFRB, CGM, CGF, PNM and SFM using IC method and to compare the measured values to those predicted from published equations.

## MATERIALS AND METHODS

The experimental protocol used in this study was approved by the Institutional Animal Care and Use Committee of China Agricultural University (Beijing, China).

### Animals, diets and experimental design

Twelve growing barrows (Duroc×Large White×Landrace, initial body weight [BW] = 32.4±3.3 kg) were used in this experiment conducted at the FengNing Swine Research Unit of China Agricultural University (Hebei, China). The 12 growing barrows were allotted to a repeated 3×6 Youden Square Design with 3 periods and 6 diets in each square. There were 6 pigs and 6 open-circuit respiration chambers [12] used in each Youden square resulting in 6 replicates for each treatment. Diets included a corn-soybean meal based basal diet and 5 test diets containing 29.25% FFRB, 29.25% CGM, 24.38% CGF, 19.50% PNM, or 29.25% SFM added at the expense of corn and soybean meal (Table 2). The inclusion levels of the five ingredients used in this experiment were based on inclusion levels used in previous experiments [1,2,4,5].

During each period, pigs were individually housed in metabolism crates for 16 d, which included 7 days to adapt to the feed, metabolism crate and environmental conditions. On d 8, the pigs were transferred to the open-circuit respiration chambers for measurement of daily O_2_ consumption and CO_2_ and CH_4_ production. During this time, pigs were fed one of the 6 diets at 2.0 MJ ME/kg BW^0.6^/d; this rather low feeding level results from the adjustment of energy intakes on the feed intake of CGM, CGF, and PNM diets for which the 2.3 MJ objective could not be achieved in a preliminary trial. Total feces and urine were collected and daily heat production (HP) was measured from d 9 to 13. On d 14 and 15, pigs were fed at maintenance energy requirement [12] (ME_m_ = 890 kJ ME/kg BW^0.6^/d) in order to adapt from the fed to the fasted state. The HP was also measured at this low feed level, but the results are not included in the present paper. On the last day of each period (d 16), pigs were fasted and fasting heat production (FHP) was determined as the HP measured during the last 8 hours of d 16 from 22:30 (d 16) to 06:30 (d 17). The FHP period started 31 h after the last meal and on animals kept in the dark to minimize physical activity.

A standard corn-soybean meal diet was fed to pigs before the experiment and during the 2 days between each test period. Pigs were fed equal sized meals twice daily at 08:30 and 15:30 and had free access to water via a low-pressure nipple drinker throughout the trial. The chambers were opened for approximately 1 hour per day at 08:30 and 15:30 h to feed pigs and collect feces. The O_2_ consumption and CO_2_ and CH_4_ production during this time were not included in the calculation of daily HP. The concentration of CO_2_ in the chamber increased when the door was closed. The calculation of HP began when the concentration of CO_2_ in the chamber was above 2,000 ppm [13]. Pigs were weighed on d 1, 9, 14, 15, and 16.

### Sample collection

During d 9 to 13, feed refusals and spillage were collected twice daily, dried and weighed. Total but separate collections of feces and urine were conducted according to the methods described by Li [13]. Feces were collected twice daily at 08:30 and 15:30 h when the chamber door was opened and immediately stored at −20°C. Urine was collected each morning at 08:30 h for each pig from plastic buckets containing 50 mL of 6 N HCl and filtered through cotton gauze. The total urinary volume produced by each pig was measured and 5% of the daily urinary excretion was stored at −20°C. At the end of the urinary collection, urine samples were thawed, and thoroughly mixed, and a sub-sample was saved for analysis. Urine was collected separately during the 24 h fasting state to calculate urinary N losses for the calculation of FHP. At the end of the experiment, fecal samples were thawed, mixed, weighed, and sub-samples were oven-dried for 72 h at 65°C. The feed and fecal samples were ground through a 1-mm screen prior to chemical analysis.

### Chemical analysis and calculations

Ingredients, diets and feces were analyzed for dry matter (DM), crude protein (CP), ash, and ether extract (EE) [14,15]. Crude fiber (CF), neutral detergent fiber (NDF), and acid detergent fiber (ADF) were determined using filter bags and fiber analyzer equipment (Fiber Analyzer, Ankom Technology, Macedon, NY, USA) following a modification of the procedure of Van Soest [16]. The gross energy (GE) in the five ingredients, diets, feces, and urine samples were analyzed using an isoperibol calorimeter (Parr 6300 Calorimeter, Moline, IL, USA) with benzoic acid as a standard. The 5 ingredients and diets were also analyzed for total starch by the glucoamylase procedure [14] while total dietary fiber in the 5 ingredients were analyzed according to Prosky [17].

The ingredients and diets were hydrolyzed with 6 N HCl at 110°C for 24 h and analyzed for 15 amino acids using an Amino Acid Analyzer (Hitachi L-8900, Tokyo, Japan). Methionine and cysteine were determined as methionine sulfone and cysteic acid after cold performic acid oxidation overnight and hydrolyzing with 7.5 N HCl at 110°C for 24 h using an Amino Acid Analyzer (Hitachi L-8800, Japan). Tryptophan was determined after LiOH hydrolysis for 22 h at 110°C using high performance liquid chromatography (HPLC) (Agilent 1200 Series, Santa Clara, CA, USA).

The DM intake from d 9 to 13 in each period was calculated as the product of feed intake and DM content of diets. GE intake was calculated as the product of the GE content of the diet and the actual feed DM intake over the 5-d collection period from d 9 to 13. The energy lost in feces, urine and methane was measured for each animal on a given diet. The ME included energy lost as urine and methane. Energy lost as methane was calculated using the 39.54 kJ/L conversion factor [18].

During d 9 to 13 of each period, O_2_, CO_2_, and CH_4_ concentrations in both ingoing and outgoing air, as well as outgoing air flow rates, were measured at 5 min intervals. These data were then used to calculate O_2_ consumption and CO_2_ and CH_4_ production during each 5 min interval and these values were averaged and extrapolated to a 24 h period. Total HP was then calculated for each day from gas exchanges and urinary loss of N according to Brouwer [18] using the following equation:

$$\text{HP​}\;(\text{kJ})=16.18\times {\text{O}}_{2}\;(\text{L})+5.02\times {\text{CO}}_{2}\;(\text{L})-2.17\times {\text{CH}}_{4}\;(\text{L})-5.99\times \text{urinary N }(\text{g})$$

Retention of energy (RE) was calculated according to following equation [7]:

RE (MJ/kg)=ME intake (MJ/d)-HP (MJ/d)

Retention of energy as protein (REP) was calculated as N retention (g)×6.25×23.86 (kJ/g). Retention of energy as lipid (RE_L_) was calculated as the difference between RE and RE_P_.

The FHP was calculated based on the equation for HP with gas concentrations and air flow obtained from only the last 8-h HP measurement on d 16. In order to base production using the same time span as used for HP, the 8-h HP was extrapolated to a 24-h period. The 750 kJ/kg BW^0.6^/d FHP value from literature data [7] was also used in calculations for comparisons.

Net energy of each diet was calculated according to Noblet et al [7] using the following equation:

NE (kJ/kg DM)=[RE (kJ/d)+FHP (kJ/d)]/DM intake (kg/d)

The average net energy value of diets and ingredients were also calculated according to the following 4 published prediction equation [7]:

$$\begin{array}{l} \text{NE }(\text{kJ}/\text{kg DM})=0.843\times \text{DE}-463/1,000\times 4.184\\ \text{NE }(\text{kJ}/\text{kg DM})=0.700\times \text{DE}+(1.61\times \text{EE}+0.48\times \text{Starch}-0.91\times \text{CP}-0.87\times \text{ADF})/1,000\times 4.184\\ \text{NE }(\text{kJ}/\text{kg DM})=0.870\times \text{ME}-442/1,000\times 4.184\\ \text{NE }(\text{kJ}/\text{kg DM})=0.726\times \text{ME}+(1.33\times \text{EE}+0.39\times \text{starch}-0.62\times \text{CP}-0.83\times \text{ADF})/1,000\times 4.184 \end{array}$$

The DM of ingredients was measured at the preparation of diets in order to calculate the DM ratio of each test ingredient in the diet. The DM of minerals and vitamins in the basal diet was 2.8% and considered to not supply any energy; therefore, the DE, ME, and NE of the basal diet was divided by 0.972 (the DM ratio of corn plus soybean meal in the basal diet) in order to calculate the DE, ME, and NE of the corn and soybean meal mixture. The difference method [19] was used to calculate the average GE, DE, ME, and NE contributions of each ingredient from the mean GE, DE, ME, and NE contents of each diet. The DE/GE, ME/DE, and NE/ME ratios could then be calculated for each ingredient and used to estimate the final DE, ME, and NE values. The DE in each ingredient was calculated as GE measured in each ingredient times the ratio DE/GE, in this case, the ME and NE in each ingredient could be calculated similarly. All calculations were done on a DM basis. The respiratory quotient was calculated as the ratio between CO_2_ production and O_2_ consumption. The apparent total tract digestibility (ATTD) of nutrients in diets was calculated according to the methods of Adeola [19]. In addition to IC measurements, NE (MJ/kg DM) of the 5 ingredients and the 6 diets were calculated using the published prediction equations.

### Statistical analysis

Diets data were subjected to analysis of variance using the Proc general linear model procedure of SAS (SAS Inst. Inc., Carry, NC, USA) with diet, period and chamber as fixed effects. The LSMEANS procedure was used to calculate mean values and PDIFF was used to separate means. In all analyses, the differences were considered significant if p<0.05.

## RESULTS

### Chemical composition of ingredients and diets

The chemical composition of ingredients and diets are shown in Tables 1 and 2, respectively. The PNM and SFM have a higher CP content than other three ingredients. The contents of EE and starch in FFRB were the highest among the five ingredients. The two corn-products (CGM and CGF) and SFM contained more TDF and NDF than FFRB and PNM. However, SFM contained almost two times more ADF and CF than the two corn-products. The analyzed contents of most amino acid (AA) in PNM were the highest among the assay ingredients, followed by SFM. The contents of Ile and Thr in FFRB were lower than in the other ingredients. The CGF contained the lowest Lys and Trp levels. The composition of the five test diets is consistent with differences in ingredients composition with the highest CP content in PNM and SFM diets, the highest starch content in FFRB diet and the highest NDF content in corn-products diets and SFM diet. The FFRB diet contained two times more EE than the other diets.

### Energy and nitrogen utilization of diets

The effects of diets and period on digestibility coefficients and energy and nitrogen balances of growing pigs are shown in Table 3. The effect of chamber on all measured variables was not significant (p = 0.15 to 0.98) and is ignored in the following sections. Most ATTD of nutrients were not affected by the period; only ATTD of CP increased with BW or age of pigs (p<0.01). The ATTD of DM and GE values were significantly affected by diet composition with the highest (p<0.01) values observed for the basal diet and PNM diet. The ATTD of CP were no different for most diets (range: 85.8% to 89.6%), except in the CGM diet where it was lower (p<0.01). The ATTD of ADF in SFM diet was the lowest among the 6 diets (p<0.01). The ATTD of EE in FFRB and SFM diets was the greatest among the 6 diets (p<0.01), followed by PNM diet.

Nitrogen intake and urinary output increased with BW of pigs (p<0.05). The urinary nitrogen output was the highest in the higher protein diets (especially in PNM diet, p<0.01). Consequently, urinary energy as a percentage of DE was the highest (p<0.05) in the PNM diet with a subsequent lower (p<0.05) ME to DE ratio. Nitrogen retention did not differ between the 6 diets and averaged 25.4 g/d. Methane energy averaged 0.7% of DE and was not affected by diet composition.

Despite no difference in ME intake (kJ/kg BW^0.6^/d), HP decreased and RE_L_ and respiration quotient (RQ) increased (p<0.01) with BW increase. There was no significant difference in HP between diets despite significant differences in ME intake. Pigs fed the diet containing FFRB had the highest (p< 0.05) RE_L_, while the pigs fed the SFM diet had the lowest (p< 0.05) RE_L_. RQ was not affected by diet and was markedly lower during the FHP period compared to fed state (0.81 vs 1.07). FHP averaged 776 kJ/kg BW^0.6^/d and was not affected by diet composition and tended to be higher (p = 0.08) during the first period. There was no significant difference in NE to ME ratio among the 6 diets (76.1% on average). The lower (p<0.01) energy values (DE, ME, and NE) were observed in the high fiber diets (corn-products and SFM diets). The other diets had rather comparable energy values.

### Apparent total tract digestibility of nutrients and energy content for ingredients

Apparent total tract digestibility of nutrients and energy content of the five ingredients are shown in Table 4. The ATTD of CP of the 5 ingredients was quite variable with the lowest value for CGM (68%); other values ranged between 79% and 89%. The ATTD of NDF or ADF in FFRB, CGF, and SFM was lower than in CGM and PNM. Finally, as a consequence of differences in dietary fiber content and its low digestibility, the ATTD of GE was the lowest in the two corn co-products and SFM that also contained the highest dietary fiber levels.

The PNM had the lowest ME to DE ratio (84.5%) while the FFRB had the greatest ME to DE ratio (95.2%). The NE to ME ratio for the five ingredients ranged between 67.2% (SFM) to 78.5% (CGF). Finally, the DE, ME, and NE values were the highest for PNM (16.90, 14.28, and 10.75 MJ/kg DM) and FFRB (16.63, 15.83, and 12.33 MJ/kg DM) and the lowest in the CGM (12.95, 12.12, and 8.75 MJ/kg DM), CGF (11.13, 9.56, and 7.51 MJ/kg DM) and SFM (10.82, 9.66, and 6.49 MJ/kg DM).

## DISCUSSION

The chemical compositions of the five ingredients were within the range of values of previous reports [1–10], however, the concentrations of NDF and ADF in FFRB were slightly less than the values published in Sauvant [20] and NRC [21]. The CGM used in the current experiment contained greater starch (19.54% vs 15.76% DM basis) and total dietary fiber (53.45% vs 46.13% DM basis) than NRC [21]. The AA values were in the range of previous reports [20,21].

The growing barrows used in the current experiment were used over 3 successive periods with 18 days per period. Therefore, the BW of pigs increased significantly over successive periods. In agreement with Noblet [22], the increase in BW was associated with increases in digestibility coefficients of nutrients and energy but it was significant only for digestibility coefficients of CP, which may be attributed to the a relatively limited BW change (39.1 to 60.1 kg). Further, NRC [21] reported that the differences in digestive utilization of diets in pigs between 25 to 50 kg would be minimal.

In current study, the ME and NE contents also increased (p<0.05) as the BW of pigs increased but without significant changes in energy digestibility and ME/DE and NE/ME ratios. The NE to ME ratio of diets was not affected by the stage of growth which agrees with the study of Noblet [23] conducted over a wider BW range (40 to 150 kg).

As in other studies [24,25], energy digestibility coefficients of the diets were lowest in the high fiber diets in connection with a poor fecal digestibility of dietary fiber in pigs. In current study, the ATTD of GE was the lowest in the two corn co-products and SFM that also contained the highest dietary fiber levels, especially SFM in which dietary fiber is particularly indigestible because of its higher degree of lignification [25]. The ATTD of NDF and ADF was higher in CGM and PNM than in the other three ingredients in this experiment, because it has been reported that extrusion may enhance soluble fiber content in diets or ingredients [26]. Bindelle et al [27] reported that the soluble fiber in pig diets was correlated with enhanced bacterial activity in the large intestine that increased in fiber digestibility. Although SFM is the by-product of the oil extraction process, the ATTD of NDF and ADF were lower than that of CGF and PNM, this result can be explained by the SFM containing the highest ADF level among the 5 ingredients.

In agreement with Noblet and Perez [28], the ME to DE ratio varied with the dietary nitrogen content. The ME to DE ratio was the lowest in the PNM diet which also contained the highest nitrogen in the current experiment. The methane energy measured in our study is consistent with data obtained in similar BW pigs [12].

There are differences in methodologies used to measure FHP. For instance, Noblet [7] measured HP on each pig at 2 feeding levels consecutively. FHP (750 kJ/kg BW^0.6^/d) was then calculated by regression of HP on ME intake and extrapolation to zero feed intake (320 measurements from 41 diets). In the current experiment, the individual FHP (average: 776 kJ/kg BW^0.6^/d) was measured directly after a period of feed deprivation of 31 h. The value was remarkably close to the estimate of Noblet [7] and to the estimates determined in our lab in a subsequent trial [12,13]. All these estimates are above those obtained by regression of HP on ME intake with animals fed at variable feeding levels which underestimates FHP [29].

The DE and ME values of the 5 ingredients measured in the current experiment were within the range of values obtained by previous studies [1,2,4,5]. The NE content of FFRB was greater than the value obtained by Sauvant [20] and NRC [21] since there was a higher EE and lower fiber content in the FFRB used in the present experiment than used by Sauvant [20] and NRC [21]. The NE content of FFRB also was greater than a blended product of hemp hulls with pea (12.33 vs 10.03) [9] that is similar to FFRB, because of FFRB has a relatively high level of starch (32.92 vs 10.90). The NE content in CGM was similar to the value of Sauvant [20] and NRC [21]. The NE content in CGF was similar to the value of Sauvant [20] but lower than in NRC [21] in connection with the lower EE and starch and higher fiber content than NRC [21]. The NE content in CGM and CGF also were similar to the NE value of wheat bran (8.75 and 7.51 vs 7.78) [30] in connection with similar EE and starch and fiber content. The PNM in our experiment contained higher CP, EE, and starch than the values published in NRC [21], which led to the DE, ME, and NE content of PNM being greater than NRC [26]. However, the NE content of PNM was lower than soybean meal (10.75 vs 11.34) [13] in connection with the higher CP content than soybean meal. Moehn et al [31] reported that an increase in dietary crude protein content results in a decreased NE content of diet. The NE content in SFM was similar to the value of NRC [21]. Comparing the five ingredients, the NE in FFRB and PNM were higher than other three ingredients in connection with the lower fiber content than CGM, CGF, and SFM. The NE in FFRB higher than PNM, because of FFRB have higher EE and starch content than PNM. The results indicated that the NE content was affected by fiber, EE and starch content in ingredients.

The NE value of the 6 diets obtained from individually measured FHP (average: 776 kJ/kg BW^0.6^/d) was slightly higher than the values obtained with the FHP estimate of Noblet [7] (750 kJ/kg BW^0.6^/d). This observation emphasizes the fact that estimation of NE for maintenance (or FHP) influences directly the absolute NE value of a feedstuff. Therefore, caution should be used when comparing the measured values with predicted values from literature equations (Table 5). To further illustrate this point, the NE value of the 6 diets and 5 ingredients was calculated according to the published prediction equations based on DE and ME (NE1). The NE values of the 6 diets calculated according to individual FHP were slightly greater than predicted from published prediction equations (0.27 MJ/kg DM), this difference becomes 0.13 MJ/kg DM when a FHP of 750 kJ/kg BW^0.6^/d is used for recalculating our NE values. This difference between measured NE values and those calculated according to the prediction equations is also illustrated by the slightly greater NE to ME ratio obtained in our study on 6 diets than the corresponding value on the data of Noblet [7] on 61 diets (76.1% vs 73.9%, on average).

The NE equations proposed by Noblet et al [7] were evaluated for their applicability to ingredients with quite variable chemical characteristics [32] and they discriminate the ingredients used in the present study (Table 5). The determined NE was, on average, 3.6% greater than the average predicted NE from 4 prediction equations, which indicates that prediction equations can well predict the NE of ingredients. However, the determined NE of CGF was 10.6% greater than the predicted NE, in addition, the difference between measured and calculated NE values from one prediction equation was greater than from the other 4 prediction equations. There is no convincing explanation for this discrepancy between measured and calculated NE values. The impact of a higher FHP value in the present trial is emphasized when the difference method is used for calculating the energy value of ingredients that are included at rather low levels. In addition, all errors of measurements or bias (effects of physical activity, etc.) are concentrated on the tested ingredient value.

## CONCLUSION

The NE determined by IC method was 12.33, 8.75, 7.51, 10.75, and 6.49 MJ/kg DM for FFRB, CGM, CGF, PNM, and SFM respectively. The FHP averaged 776 kJ/kg BW^0.6^/d and was not affected by diet characteristics. The NE to ME ratios ranged from 67% to 78%. The NE contents and NE/ME ratios of diets as measured in the present study are quite comparable to the corresponding values calculated from literature prediction equations; however, our study illustrates the complexity of measuring NE values of diets, especially in connection with the estimation of FHP values. This situation may even become more critical for ingredients that differ widely from standard diets in terms of chemical composition. Measurements of NE values of ingredients should then be done quite carefully. The use of literature prediction equations is also a suitable alternative.

## Figures and Tables

**Table 1:** Chemical composition of the five ingredients evaluated in the experiment (%, DM basis)1)

Item	FFRB	CGM	CGF	PNM	SFM
Dry matter	89.19	92.29	91.25	90.95	89.26
Chemical composition					
Crude protein	15.30	21.53	23.05	52.63	32.49
Ether extract	16.04	2.07	2.34	1.90	1.93
Starch	32.92	19.54	14.85	15.23	4.91
Total dietary fiber	20.86	53.45	44.12	16.90	45.14
Neutral detergent fiber	17.78	50.30	42.27	18.87	43.51
Acid detergent fiber	7.38	14.38	12.70	7.43	28.97
Crude fiber	6.13	12.20	11.55	5.02	25.05
Ash	8.11	1.85	5.70	6.85	8.51
Calcium	0.12	0.02	0.15	0.43	0.32
Total phosphorous	1.80	0.48	0.89	0.78	0.88
Gross energy (MJ/kg)	20.61	19.31	18.58	19.17	19.13
Indispensable AA					
Arginine	1.13	1.26	0.76	5.61	2.41
Histidine	0.50	0.73	0.80	1.34	0.85
Leucine	1.15	1.93	2.95	3.54	2.20
Isoleucine	0.53	0.73	0.67	1.65	1.20
Lysine	0.80	1.00	0.69	1.64	1.20
Methionine	0.37	0.51	0.43	0.65	0.71
Phenylalanine	0.75	0.88	0.68	2.68	1.51
Threonine	0.61	0.86	0.89	1.46	1.18
Tryptophan	0.18	0.20	0.08	0.49	0.35
Valine	0.79	1.13	1.12	1.91	1.41
Dispensable AA					
Alanine	0.99	1.35	1.91	2.22	1.51
Aspartate	1.43	1.51	1.15	6.11	3.00
Cystine	0.36	0.42	0.59	0.73	0.54
Glutamine	2.14	3.10	3.69	9.86	6.26
Glycine	0.82	1.09	1.01	2.99	1.86
Proline	0.70	1.29	2.22	2.25	1.42
Serine	0.69	1.00	1.00	2.37	1.38
Tyrosine	0.59	0.70	0.51	2.10	0.80

DM, dry matter; FFRB, full-fat rice bran; CGM, corn germ meal; CGF, corn gluten feed; PNM, solvent-extracted peanut meal; SFM, dehulled sunflower meal; AA, amino acid.

1) All samples were analyzed in duplicate.

**Table 2:** Ingredients and chemical composition of the experimental diets1)

Diets	Basal diet	FFRB	CGM	CGF	PNM	SFM
Ingredients (%)						
Corn	72.50	50.75	50.75	54.37	58.00	50.75
Soybean meal	25.00	17.50	17.50	18.75	20.00	17.50
Full-fat rice bran	0.00	29.25	0.00	0.00	0.00	0.00
Corn germ meal	0.00	0.00	29.25	0.00	0.00	0.00
Corn gluten feed	0.00	0.00	0.00	24.38	0.00	0.00
Peanut meal	0.00	0.00	0.00	0.00	19.50	0.00
Sunflower meal	0.00	0.00	0.00	0.00	0.00	29.25
Dicalcium phosphate	0.90	0.90	0.90	0.90	0.90	0.90
Limestone	0.75	0.75	0.75	0.75	0.75	0.75
Salt	0.35	0.35	0.35	0.35	0.35	0.35
Vitamin and mineral premix2)	0.50	0.50	0.50	0.50	0.50	0.50
Analyzed composition (% DM basis)						
Dry matter	86.47	87.42	88.23	87.64	87.49	87.47
Crude protein	19.70	18.50	20.30	20.30	27.20	22.90
Ether extract	3.00	6.30	3.10	2.70	2.70	2.60
Starch	51.40	47.60	35.80	41.00	43.10	37.30
Neutral detergent fiber	11.20	12.50	24.30	18.90	12.90	21.00
Acid detergent fiber	3.80	4.40	7.00	5.90	4.20	11.00
Crude fiber	3.20	3.60	6.10	5.10	3.30	9.10
Ash	5.26	6.51	4.71	5.67	5.68	6.76
Indispensable AA (% DM basis)						
Arginine	1.12	1.10	1.21	1.06	2.01	1.46
Histidine	0.58	0.51	0.62	0.65	0.74	0.66
Leucine	1.90	1.57	1.96	2.18	2.27	2.05
Isoleucine	0.79	0.66	0.78	0.79	0.95	0.88
Lysine	1.04	0.89	1.03	1.02	1.15	1.06
Methionine	0.36	0.37	0.40	0.37	0.42	0.46
Phenylalanine	1.11	0.93	1.07	0.92	1.43	1.22
Threonine	0.79	0.67	0.82	0.86	0.91	0.89
Tryptophan	0.20	0.19	0.19	0.17	0.26	0.25
Valine	0.89	0.80	0.97	0.97	1.09	1.01
Dispensable AA (% DM basis)						
Alanine	1.11	1.00	1.20	1.35	1.34	1.22
Aspartate	1.91	1.66	1.82	1.62	2.80	2.23
Cystine	0.35	0.35	0.37	0.37	0.41	0.40
Glutamine	3.48	2.91	3.48	3.81	4.89	4.36
Glycine	0.80	0.74	0.90	0.88	1.22	1.10
Proline	1.19	1.03	1.31	1.52	1.44	1.29
Serine	0.94	0.80	0.97	1.03	1.26	1.09
Tyrosine	0.82	0.74	0.79	0.73	1.03	0.73

FFRB, full-fat rice bran; CGM, corn germ meal; CGF, corn gluten feed; PNM, solvent-extracted peanut meal; SFM, dehulled sunflower meal; DM, dry matter; AA, amino acid.

1) All samples were analyzed in duplicate.

2) Vitamin-mineral premix supplied the following per kg of diet: vitamin A, 5,512 IU; vitamin D_3_, 2,200 IU; vitamin E, 30 IU; vitamin K_3_, 2.2 mg; vitamin B_12_, 27.6 μg; riboflavin, 4 mg; pantothenic acid, 14 mg; niacin, 30 mg; choline chloride, 400 mg; folic acid, 0.7 mg; thiamine, 1.5 mg; pyridoxine, 3 mg; biotin, 44 μg; Mn (MnO), 40 mg; Fe (FeSO_4_·H_2_O), 75 mg; Zn (ZnO), 75 mg; Cu (CuSO_4_·5H_2_O), 100 mg; I (KI), 0.3 mg; Se (Na_2_SeO_3_), 0.3 mg.

**Table 3:** Effect of diets and period on digestibility coefficients and energy and nitrogen balances of growing pigs1),2)

Item	Diets						Periods			RSD	p-value	
		
	Basal diet	FFRB	CGM	CGF	PNM	SFM	1	2	3		Diets	Period
BW (kg)	49.2	49.7	50.1	48.2	48.5	48.0	39.1^C^	47.6^B^	60.1^A^	5.0	0.97	<0.01
DM intake (kg/d)	1.35	1.42	1.47	1.40	1.38	1.45	1.24^C^	1.40^B^	1.60^A^	0.50	0.35	<0.01
Digestibility coefficients (%)												
DM	89.7^a^	85.8^b^	83.5^c^	82.2^c^	88.7^a^	79.3^d^	84.5	84.9	85.2	1.6	<0.01	0.51
CP	89.6^a^	87.0^ab^	82.6^c^	87.0^ab^	89.3^a^	85.8^b^	84.9^B^	87.5^A^	88.2^A^	2.0	<0.01	<0.01
NDF	62.2^a^	51.1^b^	65.8^a^	44.0^b^	62.7^a^	44.4^b^	55.6	54.9	57.0	7.2	<0.01	0.77
ADF	66.0^a^	50.4^b^	66.0^a^	47.2^b^	62.1^a^	36.3^c^	53.9	54.6	57.9	6.1	<0.01	0.26
EE	62.7^c^	73.3^a^	51.1^e^	55.5^d^	67.6^b^	71.2^ab^	62.5	64.2	64.0	3.2	<0.01	0.38
GE	89.8^a^	86.9^b^	82.6^c^	82.3^c^	89.5^a^	79.7^d^	84.6	85.2	85.6	1.6	<0.01	0.28
Nitrogen balance (g/d)												
Intake	42.7^d^	42.2^d^	47.6^c^	45.4^cd^	58.3^a^	53.1^b^	42.0^C^	48.0^B^	54.6^A^	3.2	<0.01	<0.01
Fecal output	4.4^c^	5.4b^c^	8.1^a^	5.9^b^	6.2^b^	7.5^a^	6.3	6.0	6.5	1.0	<0.01	0.43
Urine output	12.1^b^	13.7^b^	14.2^b^	16.0^b^	25.7^a^	18.1^b^	13.5^B^	15.9^AB^	20.4^A^	6.4	0.01	0.04
Retention	26.2	23.1	25.4	23.5	26.5	27.5	22.2	26.1	27.7	6.4	0.80	0.11
Energy utilization (%)												
Urinary energy % of DE	2.4^b^	3.1^ab^	3.5^ab^	4.3^ab^	5.0^a^	4.1^ab^	4.0	3.9	3.2	1.2	0.02	0.25
Methane energy (% DE)	0.8	0.6	0.6	0.9	0.7	0.7	0.7	0.7	0.8	0.2	0.24	0.42
ME/DE	96.8^a^	96.3^ab^	96.0^ab^	94.8^ab^	94.3^b^	95.2^ab^	95.3	95.3	96.0	1.4	0.04	0.39
NE/ME	76.5	76.9	75.4	76.9	76.3	74.6	74.5	76.9	76.9	3.7	0.86	0.19
Energy balance (kJ/kg BW^0.6^/d)												
ME intake	2,068^ab^	2,134^a^	2,041^b^	1,925^c^	2,067^ab^	1,959^c^	2,024	2,033	2,041	56	<0.01	0.76
HP	1,267	1,272	1,255	1,217	1,263	1,260	1,323^A^	1,217^B^	1,226^B^	83	0.88	<0.01
REP3)	384	336	365	341	387	404	368	385	356	81	0.65	0.67
REL4)	418^ab^	525^a^	421^ab^	367^ab^	417^ab^	294^b^	332^B^	430^A^	459^A^	98	0.02	0.01
RE	801^ab^	861^a^	786^ab^	708^b^	805^ab^	699^b^	700^B^	815^A^	815^A^	88	0.03	0.01
FHP	786	789	758	772	770	778	819	751	756	78	0.98	0.08
RQ												
Fed state	1.07	1.07	1.07	1.06	1.07	1.05	1.03^B^	1.08^A^	1.09^A^	0.02	0.42	<0.01
Fasted state	0.81	0.80	0.81	0.81	0.81	0.81	0.79^C^	0.81^B^	0.83^A^	0.02	0.96	<0.01
Energy values (MJ/kg DM)												
DE	16.28^a^	16.16^a^	15.11^b^	14.79^b^	16.25^a^	14.42^c^	15.40	15.52	15.59	0.29	<0.01	0.27
ME	15.75^a^	15.56^ab^	14.50^c^	14.01^d^	15.31^b^	13.73^d^	14.67^B^	14.80^AB^	14.96^A^	0.27	<0.01	0.05
NE	12.05^a^	11.97^a^	10.94^b^	10.77^b^	11.68^a^	10.24^b^	10.93^B^	11.38^AB^	11.52^A^	0.56	<0.01	0.04

FFRB, full-fat rice bran; CGM, corn germ meal; CGF, corn gluten feed; PNM, solvent-extracted peanut meal; SFM, dehulled sunflower meal; RSD, residual standard deviation; BW, body weight; DM, dry matter; CP, crude protein; NDF, neutral detergent fiber; ADF, acid detergent fiber; EE, ether extract; GE, gross energy; DE, digestible energy; ME, metabolizable energy; NE, net energy; HP, heat production; RE_P_, energy retention as protein; RE_L_, retention of energy as lipid; RE, retention energy; FHP, fasting heat production; RQ, respiration quotient.

1) Values were means of six observations per treatment.

2) With different superscript within a row means significantly different (p<0.05).

3) RE_P_ (kJ/kg BW^0.6^/d) = [N intake (g) – N in feces (g) – N in urine (g)]×6.25×23.86 (kJ/g)/BW^0.6^.

4) RE_L_ = Energy retention as fat (kJ/kg BW^0.6^/d) = [RE (kJ) – energy retention as protein (RE_P_) (kJ)]/BW^0.6^.

**Table 4:** Apparent total tract digestibility (ATTD) of nutrients and energy content of the five ingredients1)

Item	FFRB	CGM	CGF	PNM	SFM
Digestibility coefficients (%)					
CP	79.5	68.5	80.4	88.9	80.3
NDF	32.6	67.6	29.7	64.1	33.9
ADF	26.9	66.0	30.2	52.5	27.0
EE	78.4	28.3	26.5	97.5	104.0
GE	80.7	67.0	59.9	88.1	56.6
Energy utilization (%)					
ME/DE	95.2	93.6	85.9	84.5	89.3
NE/ME	77.9	72.4	78.5	75.3	67.2
NE/DE	74.1	67.5	67.5	63.6	60.0
Energy values (MJ/kg DM)					
DE	16.63	12.95	11.13	16.90	10.82
ME	15.83	12.12	9.56	14.28	9.66
NE	12.33	8.75	7.51	10.75	6.49

FFRB, rice bran; CGM, corn germ meal; CGF, corn gluten feed; PNM, solvent-extracted peanut meal; SFM, dehulled sunflower meal; CP, crude protein; NDF, neutral detergent fiber; ADF, acid detergent fiber; EE, ether extract; GE, gross energy; DE, digestible energy; ME, metabolizable energy; NE, net energy.

1) n = 6.

**Table 5:** Comparison of diets NE values measured by indirect calorimetry in the present study and estimated from prediction equations (MJ/kg DM)1)

Item	Measured_1_	Measured_2_	Difference2)	NE_1_ 3)	Difference4)
Diets					
Basal diet	12.05	11.82	−0.23	11.82	−0.23
FFRB	11.97	11.75	−0.22	11.77	−0.20
CGM	10.94	10.91	−0.03	10.66	−0.28
CGF	10.77	10.62	−0.15	10.40	−0.37
PNM	11.68	11.52	−0.16	11.42	−0.26
SFM	10.24	10.15	−0.09	9.97	−0.27
Average	11.28	11.13	−0.15	11.00	−0.27
Ingredients					
FFRB	12.33	12.14	−0.19	12.22	−0.11
CGM	8.75	9.27	0.52	8.55	−0.20
CGF	7.51	7.01	−0.50	6.79	−0.72
PNM	10.75	10.82	0.07	10.51	−0.24
SFM	6.49	6.75	0.26	6.17	−0.32
Average	9.17	9.20	0.03	8.85	−0.32

NE, net energy; DM, dry matter; FFRB, rice bran; CGM, corn germ meal; CGF, corn gluten feed; PNM, solvent-extracted peanut meal; SFM, dehulled sunflower meal; FHP, fasting heat production.

1) Measured_1_ and Measured_2_ were calculated from individual FHP measured in this experiment or FHP of Noblet et al [7] (FHP = 750 kJ/kg BW^0.6^/d) respectively.

2) Difference between Measured_1_ and Measured_2_.

3) The average of 4 predicted NE from Noblet et al [7], where i) NE = 0.843 × DE – 463/1,000 × 4.184 (NE of FFRB, CGM, CGF, PNM, and SFM were 12.08, 8.98, 7.45, 12.31, and 7.18 MJ/kg DM, respectively); ii) NE = 0.700 × DE + (1.61 × EE + 0.48 × Starch – 0.91 × CP – 0.87 × ADF)/1,000 × 4.184 (NE of FFRB, CGM, CGF, PNM, and SFM were 12.53, 8.25, 6.91, 9.99, and 5.51 MJ/kg DM, respectively); iii) NE = 0.870 × ME – 442/1,000 × 4.184 (NE of FFRB, CGM, CGF, PNM, and SFM were 12.01, 8.78, 6.55, 10.66, and 6.64 MJ/kg DM, respectively); and iv) NE = 0.726 × ME + (1.33 × EE + 0.39 × starch – 0.62 × CP – 0.83 × ADF)/1,000 × 4.184 (NE of FFRB, CGM, CGF, PNM, and SFM were 12.27, 8.18, 6.27, 9.10, and 5.35 MJ/kg DM, respectively).

4) Difference between Measured_1_ and NE_1_.
